# A Generative Model to Embed Human Expressivity into Robot Motions

**DOI:** 10.3390/s24020569

**Published:** 2024-01-16

**Authors:** Pablo Osorio, Ryusuke Sagawa, Naoko Abe, Gentiane Venture

**Affiliations:** 1 Department of Mechanical Systems Engineering, Faculty of Engineering, Tokyo University of Agriculture and Technology, Koganei Campus, Tokyo 184-8588, Japan; ryusuke.sagawa@aist.go.jp; 2 CNRS-AIST JRL (Joint Robotics Laboratory) IRL, National Institute of Advanced Industrial Science and Technology (AIST), Tsukuba 305-8560, Japan; venture@g.ecc.u-tokyo.ac.jp; 3 Naver Labs Europe, 38240 Meylan, France; naoko.abe1@naverlabs.com; 4 Department of Mechanical Engineering, Graduate School of Engineering, The University of Tokyo, Hongo Campus, Tokyo 113-8654, Japan

**Keywords:** human–robot interaction, human-centered robotics, human-in-the-loop, human factors

## Abstract

This paper presents a model for generating expressive robot motions based on human expressive movements. The proposed data-driven approach combines variational autoencoders and a generative adversarial network framework to extract the essential features of human expressive motion and generate expressive robot motion accordingly. The primary objective was to transfer the underlying expressive features from human to robot motion. The input to the model consists of the robot task defined by the robot’s linear velocities and angular velocities and the expressive data defined by the movement of a human body part, represented by the acceleration and angular velocity. The experimental results show that the model can effectively recognize and transfer expressive cues to the robot, producing new movements that incorporate the expressive qualities derived from the human input. Furthermore, the generated motions exhibited variability with different human inputs, highlighting the ability of the model to produce diverse outputs.

## 1. Introduction

Bartra [1] asserts that symbolic elements, including speech, social interactions, music, art, and movement shape human consciousness. This theory extends to interactions with society and other living beings [2], suggesting that robotic agents, as potential expressive and receptive collaborators [3], should also be integrated into this symbolic framework. However, current human–robot interactions, whether via generated voices, movement, or visual cues [4,5,6,7,8,9], are often anthropomorphized [10], leading to challenges due to unsolved problems in natural language processing [11,12] and the need for the users’ familiarization with system-specific visual cues [13]. Moreover, these systems still struggle with context understanding, adaptability, and forethought [14,15]. The ideal generalized agent capable of formulating contextually appropriate responses remains unrealized [16]. Nonetheless, the prospect of body movement could enhance these interactions.

In the dance community, body movement is acknowledged for its linguistic properties [17], from minor gestures [18] to significant expressive movements conveying intent or state of mind [19]. This expressiveness can be employed in robots to create meaningful and reliable motion [20,21,22], leveraging elements such as legibility [23], language knowledge [24], and robust descriptors [25,26]. By so doing, robots can create bonds, enhance the rapport between users and robots, persuade, and facilitate collaborative tasks [27,28,29]. Currently, however, the selection of these expressive qualities often relies on user preference or expert design [20,30], limiting motion variability and affecting the human perception of the robot’s expression [31].

In [32], the authors demonstrated the need for an explainable interaction between embodied agents and humans; furthermore, it was suggested that expressivity could hold the necessary terms for the robot to communicate its internal state effectively. Ref. [33] points out that this representation will be required for the realization of sounds and complex interactions with humans. Movement then could be the medium to realize such a system (this is further visualized in the following dance video from Boston Dynamics: https://www.youtube.com/watch?v=fn3KWM1kuAw, accessed on 20 November 2023). As discussed in [34], modeling these human factors can be accomplished using machine-learning techniques. However, direct human expressivity is often set aside in the literature, favoring definitions that could effectively be used as design guidelines for specific embodied agents or interactive technologies [35]. This leads to the question of whether or not it is then possible to rely on human expressivity and expressive movement to communicate this sense effectively. Moreover, can the robot recognize this intent and replicate the same expressive behavior to the user? The robot should communicate its internal state and do it in a manner understandable to humans. This work aims to answer these questions, exploring human expressivity transmission to any robot morphology. In doing so, the approach will be generalizable to any robot and make it possible to ascertain whether the expressive behavior contains the necessary qualities. By addressing this challenge, it is possible to enhance the human–robot interaction and open scenarios where human users could effectively modify and understand robot behavior by demonstrating their expressive intent.

Despite the availability of expressive movement descriptors, a systematic and generalized approach for generating expressive movements across various robotics embodiments and applications is required. A method that does not hinge on expert design or pre-selected qualities would increase the adaptability and versatility of robots, thereby enhancing user experience.

## 2. Related Works

### 2.1. Expressive Qualifiers

Expressive body movements are defined by low- and high-level descriptors [36]. Low-level descriptors focus on kinematics or dynamic quantities such as velocity and acceleration, whereas high-level descriptors use low-level features to describe their perceptual or semantic evaluation optimally. Notable high-level systems include Pelachaud’s qualifiers [37], Wallbot’s descriptors [38], and the Laban Movement Analysis (LMA) system, which is commonly used for dance performance evaluation [39]. The LMA system explores the interaction between effort, space, body, and shape, serving as a link between movement and language [40]. It focuses on how the body moves (body and space), its form during motion (shape), and the qualitative aspects of dynamics, energy, and expressiveness (effort). Because it quantifies expressive intent, the Effort component of LMA has been widely used in animation and robotics [41], and is utilized in this work to describe movement expressiveness.

Movements are often associated with emotions, and numerous psychological descriptors have been used to categorize body movement [42]. Scales like Pleasure–Arousal–Dominance (PAD) and Valence–Arousal–Dominance(VAD) have been used in animation and robotics [24,43,44]. However, manual selection can introduce bias [45]. While motion and behavioral qualifiers can improve user engagement with animated counterparts [46,47], no unified system effectively combines effective and expressive qualities.

### 2.2. Feature Learning

The idea of feature extraction and exploitation has seen widespread use and advancement in classifying time series across diverse domains [48,49,50]. These techniques have also been applied in image processing and natural language processing to extract meaning and establish feature connections [51,52]. Such methods have been repurposed for cross-domain applications, like the co-attention mechanism that combines image and sentence representations as feature vectors to decipher their relationships [53]. These mechanisms can analyze and combine latent encodings to create new style variations, as seen in music performances [54]. The results demonstrate that these networks can reveal a task’s underlying qualities, context, meaning, and style.

When applied to motion, the formation and generation of movement can be conducted directly in the feature or latent space, where the representation contains information about the task and any anomalies or variations [55]. Studies have shown that multi-modal signals can be similarly represented by leveraging these sub-spaces [56]. The resultant latent manifolds and topologies can be manipulated to generalize to new examples [57].

### 2.3. Style Transfer and Expressive Movement Generation

Previous research focused on style transfer using pose generation systems, aiming to generate human-like poses from human input, albeit with limitations in creating highly varied and realistic poses [58,59,60]. To address this, Generative Adversarial Networks (GAN), attention mechanisms, and transformers have been introduced, which, while improving pose generation performance, are usually confined to specific morphologies, compromising their generalizability [61,62,63].

Research suggests that a robot’s movement features can be adaptable, with human input specifying the guiding features of the robot’s motion, serving as a foundation for a divide-and-conquer strategy to learn user-preferred paths [64]. A system built on these features assists the robot’s pose generation system, showing that human motion can influence the basis functions to align with the user’s task preferences.

Although it has been shown that expressive characteristics can be derived from human movement and integrated into a robot arm’s control loop, the generated motions often lack legibility and variability [65]. In addition, much of the essence of higher-order expressive descriptors and affective qualities is lost or unmeasured. Although re-targeting can be used to generate expressive motion, it often faces cross-morphology implementation issues [66,67,68]. Burton emphasized that “imitation does not penetrate the hidden recesses of inner human effort” [40]. However, modulating motion through expert descriptors and exploiting kinematic redundancy can feasibly portray emotional characterizations, provided the motion is within the robot’s limits and the interaction context is suitable [69]. Therefore, effective expressive generation should consider both the user’s expressive intents and the task or capabilities of the robot.

## 3. Contribution

We propose a novel method for extracting expressive qualities from human movements and transferring them to different robotic structures regardless of their form. This approach, which uses a blend of supervised and unsupervised learning tasks, enables robust feature extraction and reliable transfer of expressiveness without depending on expert descriptors. It automatically identifies the essential elements of motion and integrates them into the robot’s movement. This method generates the robot’s trajectory; in this regard, it is controller-independent. The generated motion can be used with any control methodology, and the generated expressive motion can be integrated as an addition to any task-specific constraints that might be required. These constraints may include legibility, predictability, or any other qualities that might be required according to each robot task. The overarching goal of our approach is to generate an expressive robot movement that can understand and integrate the expressive qualities of human movement inputs, since any embodied behavior can help transmit these essential cues [33].

Our method can understand the expressive qualities of human movement and exploit them to generate a new movement for the robot. Unlike previous approaches where direct manipulation of the robot’s trajectory, control, and motion qualities, e.g., acceleration, velocity, and position, are the essence of the expressive definition [33,41,70,71,72], our method extracts the underlying qualities from the human, and then integrates them into the robot task, generating a new movement for the robot. This allows for a direct interaction between user and robot, and removes the need for expert design morphology-dependent constraints and the constant reprogramming seen in previous methods.

The method was tested on various robot simulations and real-world robots, including a double pendulum, mobile base, and 5 and 7 degrees of freedom (DoF) robot arms. The results showed that the generated movements mirrored human expressive feature distributions, indicating a successful expressive behavior transfer. Real-world robot experiments were verified by two Laban experts, confirming the presence of human expressiveness in the robot motions. Specifically, the expressive qualities of the double pendulum aligned with human input, and the 5DoF robot arm and mobile base showed evident changes in the Laban effort qualities.

## 4. Materials and Methods

### 4.1. Method Overview

**Figure 1:**
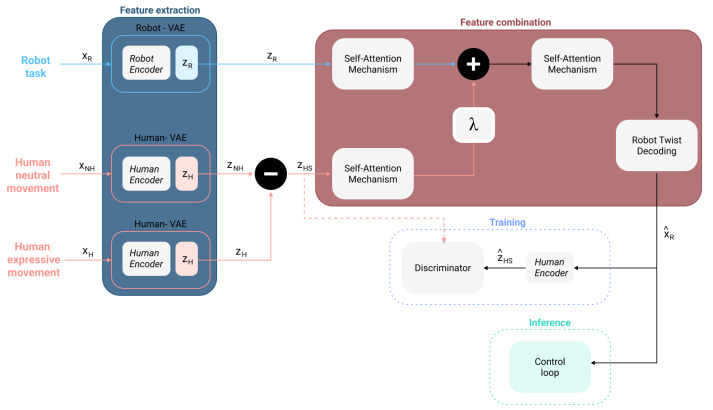
Overview of the proposed framework. Light blue highlights the components related to the robot’s task, $x_{R}$. Pink represents everything connected to human movement, $x_{H}$. Additionally to $x_{H}$, there is another input from the human: the neutral movement, which is defined as $x_{\mathrm{NH}}$. Two blocks are shown in dotted lines: one was used during the training (blue) and the other during the inference stage (turquoise). The blocks that compose the framework’s generator are feature extraction (dark blue) and feature combination (red). The latent space, i.e., the Variational Autoencoder (VAE) encoder output, of the neutral motion is represented by $z_{\mathrm{NH}}$. Simultaneously, we represent the human expressive movement latent representation as $z_{H}$, and $z_{\mathrm{HS}}$ corresponds to the latent features obtained by subtracting the neutral latent representation from the expressive latent representation. $z_{R}$ represents the latent space of the robot task, and $\hat{x_{R}}$ is the output of the generator. The new expressive robot motion has an expressive latent space denoted by $\hat{z_{\mathrm{HS}}}$, which was obtained by passing $\hat{x_{R}}$ through the human’s VAE encoder. Additionally, the parameter λ acts as an expressive gain, which can be tuned to increase or decrease the expressive content from the generated motion as required.

This study aimed to integrate human expressive qualities into robot motion using neural networks for feature extraction. The extracted features independently represent the human expressive movement and robot task. Manipulating these features allows for the creation of new robot movements with both expressive features and task-specific elements. The overall architecture of the approach is shown in Figure 1.

The method is divided into two parts: feature extraction and combination. Feature extraction condenses movement information into latent spaces using two Variational Autoencoders (VAE) [73]: one for robot tasks, represented as the linear velocities and angular velocities of an end-effector or body part of the robot, and the other for human expressive motion, derived from the acceleration and angular velocity, which were used due to the descriptive qualities of these movements [74]. The linear velocities and angular velocities of the robot provide a base representation of a robot task without requiring specific morphological knowledge. For humans, acceleration and angular velocity were chosen for their movement description.

Feature combination seeks to create a new representation of the human and robot motion features. These features are combined using independent self-attention mechanisms [51] to determine the significance of the input parts. Their outputs are additively merged (as in [54]) and processed using another self-attention layer. The decoder then reconstructs the motion as the final output of the generator.

### 4.2. Laban Effort Qualities

Throughout this work, we use the Laban Effort analysis as our base for describing and qualifying expressivity, which is why it is necessary to understand it before applying it in our method. “Effort” analysis was developed by Rudolf Laban, who investigated the dynamic structure of movement, and the expressive quality of dance [75]. The Effort includes four factors: Time, Space, Weight, and Flow. Each factor has different intensities represented in polarity; “sudden vs. sustain” in Time, “direct vs. indirect” in Space, “strong vs. light” in Weight, and “bound and free” in Flow. According to [75], each factor is described as follows: The Time factor is not about analyzing whether the movement is fast or slow. “Sudden” in Time refers to the movement that indicates a willingness to accelerate and to condense, movement in a hurry or a reaction of surprise, while “sustain” indicates a willingness to extend the time. “Direct” in the Space factor precisely addresses unidirectional orientation or focus in one direction, while “indirect” indicates movement in multiple directions. “Strong” in Weight means that the movement goes or resists gravity, while “light” refers to constant movement adjusting to gravity or diminishing the gravity effect. Flow refers to the precision and control of movement. “Bound” in Flow means that the movement is controlled, conscientious, and retrained, while “free” refers to the movement being exuberant and difficult to interrupt. These qualities can be described numerically following the descriptions proposed in [36,76]. Furthermore, this methodology has been applied to construct, evaluate, and design expressive and legible motions in robots with diverse morphologies [25,33,41,70].

### 4.3. Feature Extraction for Movement Representation in Sub-Spaces

Latent data representation is crucial for the generator. To this end, independent VAEs extract essential features from the input and reconstruct the input x as $\hat{x}=f\left(x\right)$. These VAEs encode high-dimensional data into a lower-dimensional space and then decode them back. Then, they are trained to maximize the evidence lower bound (ELBO). This maximization helps capture the intrinsic structure of the data, assuming a normal underlying latent distribution.

Each feature vector z construction involves a sequence of convolutional and Long Short-Term Memory (LSTM) layers. Different kernel sizes are used in each convolution to obtain the variations present in the data, with the first kernel capturing long-term dependencies and the subsequent kernels shorter dependencies. LSTMs encode the final feature sequence, resulting in a lower-dimensional latent space. This general structure is utilized in both VAEs for robot and human movement.

The human-motion VAE was designed to capture expressive human movement qualities, setting it apart from the robot VAE. It uses encoder features ($z_{H}$) to predict the Laban Effort qualities such as Flow, Space, Weight, and Time, which were numerically quantified as in [76]. The predictions were used in a regression task via a fully connected feed-forward neural network. Secondary tasks in optimization, as seen in [77], provided stability, regularization, and ensure feature alignment with expressiveness extraction. The final loss function is defined as follows:

(1) $$\begin{array}{c} L=E_{q_{\phi }\left(z_{H}|x_{H}\right)}\left[\log p_{\theta }\left(x_{H}|z_{H}\right)\right]-D_{KL}(q_{\phi }(z_{H}|x_{H})||p\left(z_{H}\right))+\beta \sum_{i=1}^{N}{\left(x_{{\mathrm{HLQ}}_{i}}-{\hat{x}}_{{\mathrm{HLQ}}_{i}}\right)}^{2} \end{array}$$

ELBO loss maximization involves the regular variational loss with $x_{H}$ as the human movement input and $z_{H}$ as its latent representation. The term $D_{KL}$ represents the Kullback–Leibler (KL) divergence between the estimated latent distribution and the latent prior, β is a scalar to regularize the effect of the Mean Squared Error (MSE) term coming from the Laban qualities, $q_{\phi }$ represents the encoder of the VAE (approximate posterior) with its parameters, while $p_{\theta }$ is the decoder, and $p(z_{H})$ is the latent space prior distribution. The primary goal of the first term is to reconstruct the data, whereas the KL divergence compels the model to remain in proximity to a predetermined prior. The Laban qualities loss offers regularization and forces the model to learn the most relevant features of the latent space that relate to the expressive components of the movement. This last term of the loss compares, through the MSE, the human movement Laban qualities ($x_{\mathrm{HLQ}}$) to the network’s output (${\hat{x}}_{\mathrm{HLQ}}$) for *N* samples. The robot VAE uses the same loss definition but without the Laban qualities term.

### 4.4. Adversarial Generation Implementation

In this work, we propose using an adversarial scheme to generate expressive robot motions that considers the expressive inputs from the human movement. It is shown that through this method, it is possible to learn speech and movement user-specific styles and generate new animations that reflect these features [58]. Improving upon the previous work, we aimed to expand this methodology to generate expressive robot motions that reflect user-specific expressive qualities. The adversarial method focuses on the interaction between the discriminator and generator networks. The general loss of the GAN methodology can be formulated as follows:

(2) $$\min_{G}\max_{D}E_{x}\left[\log D(x)\right]+\;E_{z}\left[1-\log D(G(z))\right]$$

Our approach partitions the job of sub-space representation and generation into two different parts. The latent representation is obtained through the VAEs, while the generation is learned through the GAN methodology. To this end, the robots’ and humans’ VAE encoders are trained separately from the general GAN framework. These VAEs are trained following the definition presented in Section 4.3. When the training for these two models is complete, they are coupled with the block from feature combination (see Figure 1). At this stage, VAE models remain static, allowing the GAN training to take place, focusing on the generation using pre-trained input representations, and ensuring stability by splitting tasks into extraction and generation. At inference time, the complete model, composed of feature extraction and feature combination (see Figure 1) is used to generate the new robot motion.

The goal of the GAN method is to minimize the generator loss while increasing the discriminator’s ability to distinguish between the real data and the generator’s output. The task of the discriminator is to identify the presence of the expressive qualities in the generated output; its loss function is defined in Equation (3). Here, $f_{HVAE}$ refers to the human VAE encoder, $f_{RVAE}$ refers to the robot encoder, and *G* and *D* refer to the generator and discriminator, respectively. The generator, *G*, will be composed of the feature extraction and feature combination blocks; refer to Figure 1. $x_{H}$ is the input with expressive content from the human movement, $x_{\mathrm{NH}}$ is the neutral human motion, and $x_{R}$ is the robot input. $z_{H}$ is then derived through $z_{H}=f_{HVAE}\left(x_{H}\right)$, $z_{\mathrm{NH}}$ is derived through $z_{\mathrm{NH}}=f_{HVAE}\left(x_{\mathrm{NH}}\right)$, and $z_{R}$ is derived through $z_{R}=f_{RVAE}\left(x_{R}\right)$. The objective of the generator is to produce expressive robot motions. The generator loss function is framed in Equation (4), improving the formulation presented in [58]. This generator loss will preserve the robot’s task while enforcing expressive output diversification. The terms α, γ, ζ, and ρ denote regularization scalars that will balance the effect of each loss term; during training, the values used were 2, 100, 10, and 15, respectively.

(3) $$L_{D}=\;E_{z_{H}}\left[\log D(z_{H})\right]+\;E_{x_{H},x_{NH},x_{R}}\left[1-\log D(f_{HVAE}\left(G\left(x_{H},x_{\mathrm{NH}},x_{R}\right)\right))\right]$$

(4) $$L_{G}=\alpha \cdot L^{MSE}+\gamma \cdot L^{style}+\zeta \cdot L^{KD}+\rho \cdot -\;Ex_{H},x_{NH},x_{R}\left[\log D(f_{HVAE}\left(G\left(x_{H},x_{\mathrm{NH}},x_{R}\right)\right))\right]$$

The MSE loss, defined in Equation (5), compares the original robot input, $x_{R}$, to the generated output, ${\hat{x}}_{R}$, ensuring the integrity of the primary task. To preserve the expressiveness and encourage input variability, a diversity regularization technique was applied [78], as shown by the style loss in Equation (6). This method amplifies the differences in human expressive representations. Each iteration employs two distinct expressive human samples: $z_{H_{1}}$ and $z_{H_{2}}$. Both $z_{H_{1}}$ and $x_{{\mathrm{NH}}_{1}}$ are the latent representation through $f_{HVAE}$ of the inputs $x_{H}$ and $z_{\mathrm{NH}}$, while $z_{H_{2}}$ and $x_{{\mathrm{NH}}_{2}}$ are the latent representations through $f_{HVAE}$ of two random samples of the human expressive and neutral motions. These random inputs should still belong to the same user as the one who realizes $z_{H_{1}}$ and $x_{{\mathrm{NH}}_{1}}$.

The Huber loss definition, $L_{\delta }$, can be seen in Equation (7). This loss function compares two inputs, y and $\hat{y}$. It follows a quadratic behavior for smaller input differences, and a linear trend for larger deviations, where δ is a threshold value to define the behavior of the loss; this parameter is used as 1.0. The KL divergence, referenced in Equation (8), is used between the latent distribution of human expressivity, $P(z_{H})$, and the generated robot’s motion latent representation, $Q({\hat{z}}_{H})$, where ${\hat{z}}_{H}$ is derived through ${\hat{z}}_{H}=f_{HVAE}\left({\hat{x}}_{R}\right)$. As the VAE components remain unchanged during training, this KL term helps optimize the attention mechanisms and generation block to align with the two latent expressive distributions.

The discriminator network, *D*, evaluates the generator’s output, $G(x_{H},x_{\mathrm{NH}},x_{R})$, for its expressivity compared to the human expressive motion input. The objective is to fool the discriminator through the generator output. The closer the generated robot motion expressive qualities are to the human’s input, the more likely the discriminator will predict these two inputs to be equally valid. To represent both the robot’s generated motion and the human expressive in a common space that the discriminator can use to evaluate their expressive intent, it was decided to make use of the human VAE encoder (see Figure 1), $f_{HVAE}$. While this encoder was not optimized for robot motions, it was capable of recognizing the expressive qualities present in the motion. The result latent spaces, $z_{H}$, for the human expressive input and $f_{HVAE}\left(G\left(x_{H},x_{\mathrm{NH}},x_{R}\right)\right)$ for the generated motions, provide the inputs to the discriminator, guiding the optimization of the objective function presented in (3).

(5) $$L^{MSE}=\sum_{i=1}^{N}{\left(x_{R_{i}}-{\hat{x}}_{R_{i}}\right)}^{2}$$

(6) $$L^{style}=-\;E\left[\frac{L_{\delta }\left(G\left(x_{R},x_{{\mathrm{NH}}_{1}},x_{H_{1}}\right),G\left(x_{R},x_{{\mathrm{NH}}_{2}},x_{H_{2}}\right)\right)}{\left|\right|z_{H_{1}}-z_{H_{2}}\left|\right|}\right]$$

(7) $$L_{\delta }=\left\{\begin{array}{cc} \frac{1}{2}{\left(y-\hat{y}\right)}^{2} & if\left|(y-\hat{y})\right|<\delta \\ \delta (\left(y-\hat{y}\right)-\frac{1}{2}\delta ) & otherwise \end{array}\right.$$

(8) $$L^{KD}=P\left(z_{H}\right)\left|\right|Q\left({\hat{z}}_{H}\right)$$

### 4.5. Neural Network Architecture Specifications

Human and robot motion data were restructured into 60-sample windows. As input for the architecture shown in Figure 1, each VAE encoder accepts a 60 × 6 time series signal. All inputs are processed to fit this input shape. The initial layer used nine 7 × 7 filters with rectification and batch normalization. The subsequent layer utilized 12 5 × 5 filters, also with rectification and normalization. Three LSTM layers of 25 units each were applied, followed by two fully connected linear output layers. The decoder is mirror-like to the encoder, with the convolutions replaced by deconvolutions and an additional linear output layer. Attention mechanisms utilize multi-head attention with six heads and an embedded dimension of 30. The twist decoding block is similar to the VAE decoder. The discriminator features three successive fully connected layers with sizes of 500, 500, and 1.

### 4.6. Training Procedure

Two datasets were employed in this study: an expressive human motion dataset and a robot motion dataset. The human dataset from [79] focuses on the walking patterns of four emotions. This dataset is made out of walking motions of four different emotions (neutral, angry, happy, and sad) for four participants. Each participant has their own neutral motion representation and emotive motion. Only the acceleration and angular velocity of the wrists were used. The robot dataset combines trajectories from two established datasets [80,81] and a custom dataset using a 7DoF robot arm for tasks such as pick-and-place. The human dataset contains 2900 samples, and the robot dataset contains 11,600 samples, with each sample being a 60 × 6 signal.

The human dataset provided expressive motions, whereas the robot dataset contributed diverse robot task examples. The model’s goal was to merge human expressiveness with robot tasks. The generator took three inputs (see Figure 1): one robot and two human inputs. Any human motion included a neutral state and an expressive motion. The neutral feature representation was subtracted from the current expressive motion latent representation to distinguish the expressivity. The neutral and expressive motion for the human was a one-to-one correspondence given to each user, meaning that at training and inference, both motions were related to the same user. The robot’s motion was randomly selected regarding the human since any robot’s movement could be modified according to the human’s expressive input. This random pairing was enforced during the GAN training by selecting a human expressive and neutral motion corresponding to the user and a random robot movement. The robot motions, the human neutral, and expressive movements were utilized both at the training and inference stages.

Both GAN and VAE training used the AdamW [82] optimizer with a variable learning rate decreasing by a factor of 10 whenever the learning stagnated. The initial learning rate was set to 0.001. The GAN model underwent 100 epochs, whereas the VAEs underwent 200 epochs, with the GAN having a 15-epoch warm-up period before adding the diversity regularization term (6). All the implementation was performed in Python using the Pytorch library [83].

## 5. Results

### 5.1. Expressive and Affective Evaluation

Using an approach similar to that in [84], the method was assessed by comparing the robot, human, and network output datasets using Laban Effort Qualities (LEQ). Kernel Density Estimation (KDE) offers insights into the LEQ distribution, enabling distance and similarity evaluations across datasets. The generated dataset integrated human data with random robot inputs to explore the robot’s response to human expressiveness. A key variable was the network’s gain, λ, which modulates human expressiveness. A high λ enhances expressivity, whereas a low value prioritizes the robot task.

To the best of our knowledge, this is the first work to address expressive transmission in general terms. Previous works [9,33,41,70,71,72] relied on expert descriptors or interactive interfaces to enact expressive and emotive behavior. This is why our experimental setup focuses on analyzing the effectiveness of transmitting human expressivity to the robot. No current benchmark exists for effective expressive transmission. The current methods that can be used are the numerical analysis of the generative capabilities of the method to align to the LEQ and the use of Laban experts to asses the expressivity transmission. Furthermore, they do not address the problem of dealing with multiple robot embodiments.

**Figure 2:**
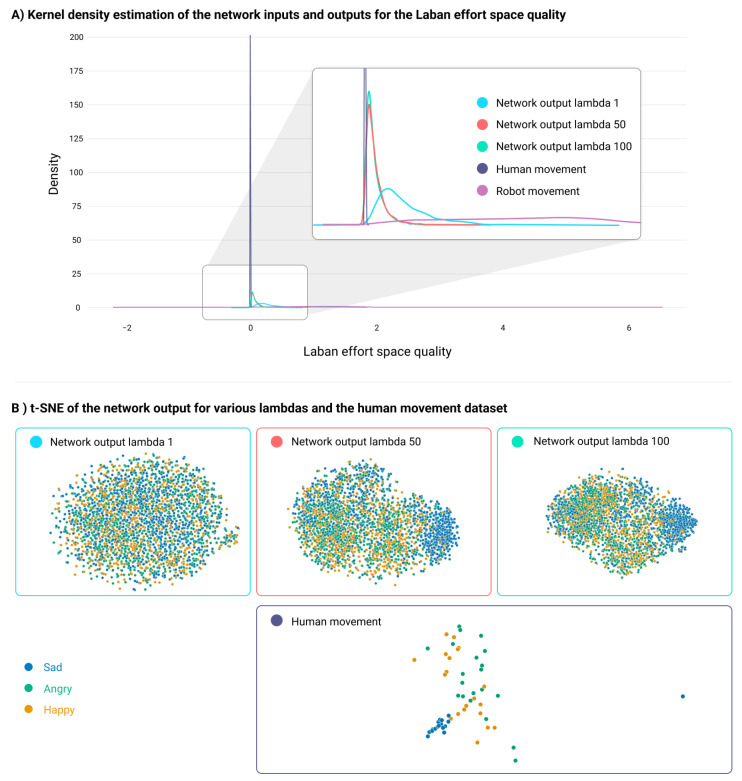
Network output distribution and representation analysis. (**A**) Kernel density of the Space Laban Effort quality for human (dark purple), robot (purple), and generated outputs at λ=1 (light blue), λ=50 (orange), λ=100 (mint green). Increasing λ makes the generated dataset more like the human, retaining robot features. (**B**) t-SNE plots of human data and network outputs at varying λ. Emotion labels: sad (blue), angry (green), and happy (yellow). With a rising λ, the sad emotion clustering becomes clearer in the generated output.

Figure 2A demonstrates that the KDE for the generated data fell between the human and robot KDEs for the LEQ Space quality at λ values of 1, 50, and 100. This indicates a shift in the robot’s mean distribution towards the human’s distribution, infusing human expressivity into robot actions. An increase in λ brought the generated output KDE close to the human KDE. The Kolmogorov–Smirnov test confirmed this by assessing whether samples from different KDEs originated from the same distribution. Under an alternate hypothesis—samples being ‘greater’ for humans and ‘less’ for robots—a *p*-value under 0.05 supports the alternate across all KDEs. This indicates that human expressivity influenced the robot dataset, nudging the generated KDE closer to the human mean.

**Figure 3:**
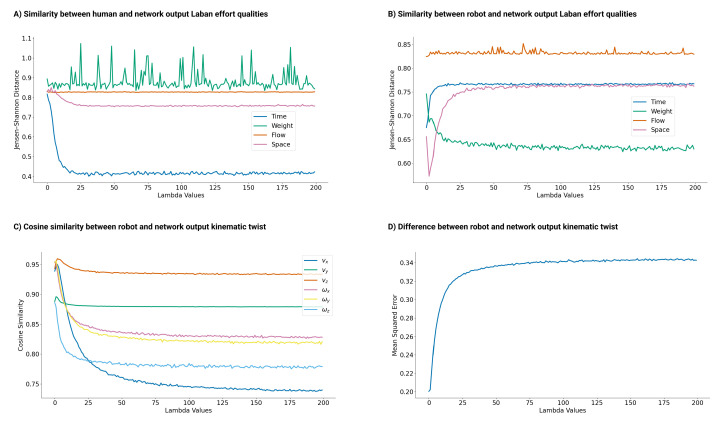
Similarity analysis. (**A**) Jensen–Shannon distance of Laban Effort qualities between generated and human datasets. As λ increases, the Time and Space qualities converge. (**B**) Jensen–Shannon distance for Laban Effort qualities between generated and robot datasets. Time and Space drift apart with increasing λ, while Flow remains stable and Weight decreases. (**C**) Cosine similarity between network output and robot motion; higher λ values diminish similarity. (**D**) The mean squared error between the network output and robot motion; increasing λ amplifies discrepancies.

The effect of varying λ values on KDE similarities was studied using the Jensen–Shannon distance (JSD) [85]. Smaller JSD values indicate a higher similarity. Figure 3A shows that increasing λ narrowed the distance between the KDEs of the generated and human dataset LEQ qualities, particularly in Time and Space. The distance between the Weight feature remained inconsistent, whereas the Flow distance plateaued after λ>10. In contrast, Figure 3B shows that the JSD between the robot and the generated KDEs increased for Time and Space but reduces for Weight. The flow feature remained at 0.83, emphasizing the trade-off between robot task behavior and human expressiveness with varying λ values.

The λ expressive trade-off impact on robot tasks was evident when assessing the alignment between the generated output and the input robot motion. Figure 3C depicts this by using cosine similarity and mean square error (Figure 3D) across λ values from 0 to 200. With increasing λ, the cosine similarity decreased, affecting the Y and Z linear velocities the least. The mean square error revealed task alterations, notably for λ between 1 and 50. The trend softened for λ>100; expressiveness remained a priority over the robot’s initial task.

To gain insights into the nuances of the affective human movements dataset, the dimensionality reduction algorithm TSNE [86] was employed. This dataset encompasses walking motions representing four emotions: sad, happy, anger, and neutral. Neutral was omitted from the analysis, as it was viewed as an extra input to the model. In the TSNE plot of human data (Figure 2B), sad movements clustered distinctly, whereas angry and happy emotions overlapped. This pattern persisted post-VAE training. It was hypothesized that the generated linear velocities and angular velocities of the robot would display a similar pattern when processed by the human VAE encoder, emphasizing affective qualities. As shown in Figure 2B, varying λ values altered the TSNE representation. With λ=1, emotions blended, but increasing λ separated the sad cluster from the angry and happy clusters, highlighting λ’s role in affective nuances. Consequently, the generated twist output reflected the inherent characteristics of the raw affective human movement dataset in latent space.

### 5.2. Simulation

**Figure 4:**
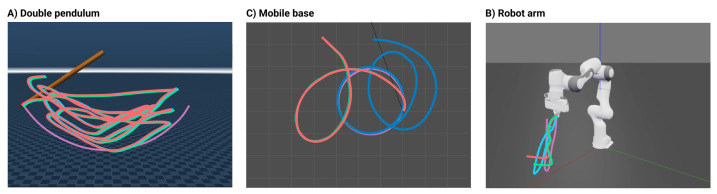
Effect of λ values in generated trajectories. Trajectories for λ=1 (light blue), λ=50 (orange), and λ=100 (mint green) on different robots; base task in purple. (**A**) Double pendulum shows no λ variation. (**B**) Robot arm modifies the task at λ=1 and loses it as λ rises. (**C**) Mobile base alters task at λ=1 and deviates more with higher λ.

The method’s adaptability was tested using a series of simulation experiments on multiple robotic platforms, with a gain λ ranging from 1 to 100. The test platforms are illustrated in Figure 4. The baseline trajectories were a continuous swing for the double pendulum, a pick-and-place task for the 7DoF robot arm, and a spatial circle for the mobile robot. Integrating the network output into movements led to discernible changes at varying λ values. All robots were simulated in Mujoco [87] and Gazebo [88], using ROS 2 [89] and the Python Robotics Toolbox [90] for controlling the robot and communicating with the simulator.

All trajectories designed for the robot reflect the usual tasks the robot might perform in a real scenario. For example, the pick-and-place task for the 7DoF robot arm is a common objective in industrial settings. However, the focus was not to prioritize the task itself, since the objective is to transmit human expressivity to robots. The idea behind having this task was to have a common scenario and understand how expressivity might be applied in this case.

For the double pendulum (Figure 4A) at λ values of 1, 50, and 100, there was more elbow joint activity, spanning a broader task space, yet the main swing remained. Interestingly, this setup displayed minor variations owing to λ, with changes mostly in amplitude and displacement.

In the case of the motion of the robot arm (Figure 4B), at λ=1, its trajectory resembled the initial motion but ended differently. At λ=50 and λ=100, the arm descended and remained at distinct end positions.

The mobile base (Figure 4C) shared similarities with the robot arm. While the task was consistent at λ=1, it changed for higher values. The motions at λ values of 50 and 100 resembled each other, which is consistent with the JSD in Figure 3A, indicating a minimal change for λ>50.

Using higher λ values emphasized the expressive trade-off, aiding the evaluation of the effects of all emotion labels from the human motion expressive dataset. This reveals how the network output may represent affective qualities.

**Figure 5:**
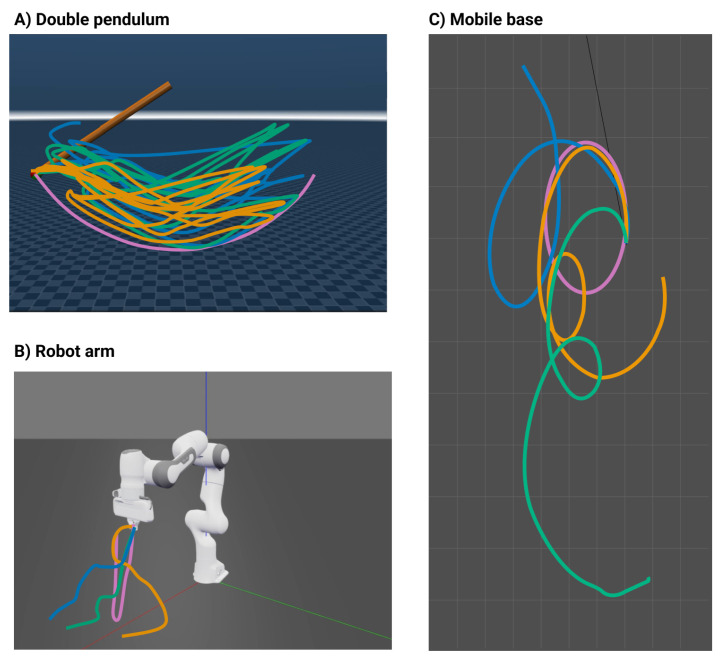
Effect of emotion labels in generated trajectories. For each emotion: sad (blue), angry (green), and happy (yellow), from the human dataset, movements were generated across morphologies, with base tasks in purple. (**A**) Double pendulum: varied trajectories by emotion. (**B**) Robot arm: similar paths, but different end positions. (**C**) Mobile base: distinct paths for each emotion, covering more task space.

Figure 5 shows the outcomes for the three simulated robots—double pendulum, robot arm, and mobile base—across emotions: anger, happiness, and sadness. The input emotions were varied by changing the human input movement with the corresponding emotion the actor performs. All trajectories used λ=100, given the stabilization of the JSD between human features and the generated output. Unlike the prior consistent behavior of the double pendulum in Figure 4A, Figure 5A shows the emotional states that distinctly impacted its movements. Although it maintained a swinging motion, their positions and the covered task space differed notably, with evident separations in outputs across emotions. This distinction persisted for the robot arm (Figure 5B).

For the robot arm, each emotional output resembled the others regarding task performance. The pick-and-place task disappeared, and the end-effector remained at the ground. However, the paths for reaching these endpoints differed. This motion, even at λ=100 with the latent space of Figure 2B, shows that emotions like sadness could still be differentiated from happiness and anger, as long as the data points were not closely situated in the latent space for all emotional states.

Regarding the mobile base (Figure 5C), sadness contrasted with happiness and anger, utilizing more task space to the left. This result mirrors the anticipated latent patterns shown in Figure 2B for λ=100. Happiness and anger favored a semi-circular path, encompassing a wider task space at the bottom of the surface; remaining intertwined. The findings highlight the method’s ability to craft varied motions based on the affective nuances of the data.

### 5.3. Real World Implementation

After the simulation, the method was tested in real-world scenarios using λ values of 1 and 100, covering all the emotion labels from the human dataset. These extremes were chosen based on prior analyses: λ=1 retained most task characteristics, while expressive effects plateaued after λ=100. The goal was to evaluate expressive traits in robot motions. Laban experts annotated the movements using the LEQ, comparing human and robot motions to assess the difference and verify the human features influencing the robot’s trajectory.

**Figure 6:**
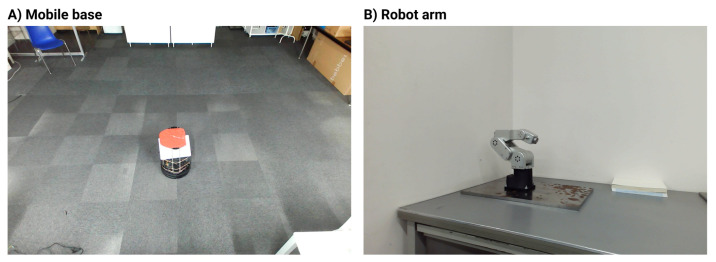
Experimental setup. Experimental setup for (**A**) the mobile base and (**B**) 5DoF robot arm.

Two platforms—a mobile base and a 5DoF robot arm—were captured in video performing tasks with human-influenced expressiveness. A total of 13 unique 30 s videos were recorded for each, including the basic task and versions altered by two λ values and three emotional states. Figure 6 shows the two experimental setups.

The robot arm traced a square in the Y-Z plane, keeping its X-axis position, while the mobile base drew a circle. These reference tasks helped us to highlight the effects of expressiveness. Similar to the simulation stage, the tasks were based on common objectives the robots might faced in the real world. However, the objective of this real-world experiment was to verify the effective transmission of the human expressive qualities to the robot embodiment, and observe whether expressive movements were generated in a real world setting. The adherence to the task or its significance were not relevant for the study with the Laban experts.

After obtaining the video recordings, two Laban experts reviewed them. They annotated the robot’s movements according to the Laban Effort qualities: Time, Flow, Weight, and Space. Each quality has opposing descriptors that can characterize a movement. Given the movement length, the experts evaluated them as a choreography. The video was divided into individual robot movements, each of which were assessed separately. The most frequent descriptor for each quality represents the movement. The Laban Effort qualities descriptors are: ‘Bound’/‘Free’ for Flow, ‘Direct’/‘Indirect’ for Space, ‘Sudden’/‘Sustained’ for Time, and ‘Strong’/‘Light’ for Weight. Although the Laban analysis follows set protocols, it is subjective. The interplay between the qualities is explained in [75]. An example of the videos analyzed by the Laban experts can be seen in Video S1. On it, the recordings for the human, double pendulum, mobile base, and robot arm can be visualized for a specific emotion.

The input human movements have qualities labeled as Free, Indirect, Sudden, and Light, representing Flow, Space, Time, and Weight. This set an expressive benchmark for the robot. Although emotional variations exist in human movements, these Laban qualities remain consistent. The qualities change to Free, Direct, Sustained, and Light in the neutral state. These qualities were derived from a Laban expert’s annotation of four videos of human walking motions: one for each emotion and one for the neutral state. The experts focused on the annotation of the arm movement.

An initial double pendulum simulation test confirmed the method’s effectiveness in producing expressive attributes. Four distinct videos were created, each showcasing the pendulum and annotated by Laban experts. These videos depict four different emotions and a base task. Given the pendulum’s resemblance to a human arm, it was anticipated that network-applied expressive qualities would align with human demonstrations. While the base task, i.e., a simple swing, symbolized a neutral emotion, the post-annotation qualities were Free, Direct, Sustained, and Light. Each emotion’s generated motion had qualities labeled as Free, Indirect, Sudden, and Light, mirroring human demonstration attributes. This consistency hints at the network’s proficiency in integrating expressive traits into generated motions.

Upon verifying the double pendulum’s expressive uniformity, its impact on other platforms was explored. The 5DoF robot arm’s motion had attributes labeled as Free, Direct, Sudden, and Light. As emotions and λ values shifted, different traits emerged. At λ=1, the attributes were Free, Indirect, Sustained, and Light across all emotions and motion variants. This mirrors descriptors related to human actions. However, with λ=100 across all emotions, the robot retained its Flow, Time, and Weight attributes—Free, Sustained, and Light. Its Space descriptor shifted to Direct, aligning with the primary task. This contradicted expectations, as a higher λ should make robot actions expressiveness more human-like. For the three motions at λ=100, Flow changed to Bound, except in the Angry emotion, which showed both Free and Bound.

For the mobile base, the robot’s circular trajectory amplitude variations did not alter the core Laban attributes, marked as Free, Direct, Sustained, and Light. This suggests robustness despite the amplitude fluctuations. Rapid robot movements did not sway the Time attribute, which upheld a consistent pace. The ‘Sustained’ descriptor underscored the robot’s potential for ongoing motion. Its on-screen actions focused mainly on spatial movement via acceleration alterations and a lack of distinct movement qualities. This might be because the human neutral state overshadows expressive attributes, given identical Laban descriptors for mobile base actions and human neutral inputs. The Laban experts highlighted that the lack of limbs may limit the expressive diversity of the mobile base, affecting comprehensive expressive quality conveyance.

Furthermore, the Laban experts pointed out the morphological differences between robots and human arms. Hence, the Weight and Flow attributes carried less significance, and Time and Space were prioritized when discerning expressiveness. This mirrors the simulation results where the Jensen–Shannon distance between generated and human movements revealed that the Weight and Flow components showed minor changes. Still, Time and Space produced smaller values (refer to Figure 3A).

## 6. Discussion

The features harnessed by the model, refined via LEQ use, adeptly altered the robot’s motion. The model’s robustness and capabilities suggest the alignment of generated motions with the expressive nuances of human movement, which is evident in the simulation and expressive validation findings. Laban expert annotations, particularly for the double pendulum and robotic arm, underscored this idea. The model maintained intrinsic data relationships even without direct emotion recognition training. Emotion interplay and the λ factor created dynamic motion, amplifying the expressiveness of robot movement. The simulation insights highlight this variety, showing that the model crafts a distinct expressive robot motion for each emotion combined with its λ. These findings hint at the model’s robust flexibility and adaptability in mirroring and transmitting expressive subtleties.

The simulation phases and real-world implementation showed trajectory variations; however, leveraging the Laban Effort qualities for motion expressivity was unsuccessful. This challenge is notable in the mobile base real-world scenarios. Trajectory modifications, such as start–end position shifts or acceleration changes, did not always translate into clear expressivity. Discrepancies surfaced when comparing the Laban qualities identified by experts in human movements and those observed in the mobile base and 5DoF robot arm. However, when the morphologies were mirrored, which was evident in the double pendulum and human arm, Laban’s qualities remained consistent. Such insights spotlight hurdles in the use of Laban annotations for diverse morphologies. Although past research indicates the successful application of Laban qualities in crafting non-humanoid motions and allowing non-experts to discern robot expressivity shifts [91], these changes can be subtle, even for seasoned experts. Overcoming this may demand refined adjustments, possibly weaving in more variables to emphasize morphological nuances or bolster expressive feature portrayal.

## 7. Conclusions

This study introduces a method for equipping robots with nuances of human expressivity. This approach effectively recognizes expressive behaviors and extracts them from the physical signals, acceleration, and angular velocities. It then combines these features with robot tasks to produce new expressive motions. When tested in both simulated and real-world environments across various robot designs, the method showcased its adaptability and broad application.

Through the Laban qualities analysis and the feedback from Laban experts, the method proved to be sufficient to understand the expressive qualities from the human motion input and transfer them to the robot’s motion. This implies that it is possible to characterize the expressive intent by relying on the acceleration and angular velocity of the human input from any body part. Moreover, it proves that modifying a robot’s expressive demeanor is feasible without requiring expert design and constant reprogramming. Its application and use with three different embodiments in a simulation and a real-world scenario showcased an effective expressive transmission with diverse robotic morphologies.

As robots become more predominant in our daily lives, especially in our homes, social spaces, and work settings, they will be required to understand, comply, and modify their demeanor according to their user’s inputs. Expressivity can work in this regard as a common trait. Our framework serves as a preliminary approach in this regard; by removing the need for specific morphology constraints, additional interfaces, or multi-modal requirements, it is possible to deliver a widely applicable interactive medium. By relying on movements, which are capabilities common to most robots nowadays, it is possible to have the same medium of interaction with the human user. Our results highlight these facts, which will enable various applications, in the arts and collaborative settings. By simply relying on movement, the behavior of the robot will be affected, and the necessary characteristics will be integrated into the robot’s behavior. In this regard, the users’ trust, user experience, and the overall interactive capabilities of the robots will be enhanced, thus providing more versatile interactions, enriched artistic expressive representations, and more explainable robot behavior.

## 8. Limitations and Future Works

Although the double pendulum results aligned with the expected expressive qualities from the human movement, the method presented difficulties when embedding the expressive qualities to morphologies that do not closely resemble the human body. This partly has to do with the analysis through the use of Laban experts since these changes are subtle, even for them. Further research will explore means of generalization through the use of direct guidance from expert feedback to train the generative model, additional constraints in the control loop of the robot, and we will perform user studies to explore their perceptions.

Even though the method was tested on various robots, exploration with humanoid robots remains challenging owing to difficulties in determining optimal input points for their full-body expressivity. Future research could investigate this issue and incorporate reinforcement learning with human feedback to enhance the model. Previous studies indicated that reinforcement learning can improve generative skills with minimal data [92]. By harnessing feedback from Laban experts, it may be possible to align closely with the Laban Effort qualities observed in human movements and match them with user preferences. Additionally, an expanded dataset for both robots and humans can further enhance the method’s feature extraction and generation capabilities.

## Data Availability

Data are contained within the article and supplementary materials.

## Supplementary Materials

The following supporting information can be downloaded at: https://www.mdpi.com/article/10.3390/s24020569/s1, Video S1: A Generative Model to Embed Human Expressivity into Robot Motions.

## Abbreviations

The following abbreviations are used in this manuscript:

LMA	Laban Movement Analysis
PAD	Pleasure–Arousal–Dominance
VAD	Valence–Arousal–Dominance
GAN	Generative Adversarial Networks
DoF	Degrees of Freedom
ELBO	Evidence Lower Bound
VAE	Variational Autoencoders
KL	Kullback–Leibler
MSE	Mean Squared Error
LSTM	Long Short-Term Memory
LEQ	Laban Effort Qualities
KDE	Kernel Density Estimation
JSD	Jensen–Shannon Distance
